# The global population structure and evolutionary history of the acquisition of major virulence factor-encoding genetic elements in Shiga toxin-producing *Escherichia coli* O121:H19

**DOI:** 10.1099/mgen.0.000716

**Published:** 2021-12-08

**Authors:** Ruriko Nishida, Keiji Nakamura, Itsuki Taniguchi, Kazunori Murase, Tadasuke Ooka, Yoshitoshi Ogura, Yasuhiro Gotoh, Takehiko Itoh, Atsushi Toyoda, Jacques Georges Mainil, Denis Piérard, Kazuko Seto, Tetsuya Harada, Junko Isobe, Keiko Kimata, Yoshiki Etoh, Mitsuhiro Hamasaki, Hiroshi Narimatsu, Jun Yatsuyanagi, Mitsuhiro Kameyama, Yuko Matsumoto, Yuhki Nagai, Jun Kawase, Eiji Yokoyama, Kazuhiko Ishikawa, Takayuki Shiomoto, Kenichi Lee, Dongchon Kang, Koichi Akashi, Makoto Ohnishi, Sunao Iyoda, Tetsuya Hayashi

**Affiliations:** ^1^​ Graduate School of Medical Sciences, Kyushu University, Fukuoka, Japan; ^2^​ Graduate School of Medicine, Kyoto University, Kyoto, Japan; ^3^​ Graduate School of Medical and Dental Sciences, Kagoshima University, Kagoshima, Japan; ^4^​ Kurume University School of Medicine, Fukuoka, Japan; ^5^​ Graduate School of Bioscience of Biotechnology, Tokyo Institute of Technology, Tokyo, Japan; ^6^​ Advanced Genomics Center, National Institute of Genetics, Shizuoka, Japan; ^7^​ Faculty of Veterinary Medicine, University of Liege, Liege, Belgium; ^8^​ Universitair Ziekenhuis Brussel (UZ Brussel), Vrije Universiteit Brussel (VUB), Brussels, Belgium; ^9^​ Osaka Institute of Public Health, Osaka, Japan; ^10^​ Toyama Institute of Health, Toyama, Japan; ^11^​ Fukuoka Institute of Health and Environmental Sciences, Fukuoka, Japan; ^12^​ Oita Prefectural Institute of Health and Environment, Oita, Japan; ^13^​ Akita Prefectural Institute of Public Health, Akita, Japan; ^14^​ Yamaguchi Prefectural Institute of Public Health and Environment, Yamaguchi, Japan; ^15^​ Yokohama City Institute of Public Health, Kanagawa, Japan; ^16^​ Mie Prefectural Institute of Public Health and Environmental Sciences, Mie, Japan; ^17^​ Shimane Prefectural Institute of Public Health and Environmental Science, Shimane, Japan; ^18^​ Chiba Prefectural Institute of Public Health, Chiba, Japan; ^19^​ Shiga Prefectural Institute of Public Health, Shiga, Japan; ^20^​ Ishikawa Prefectural Institute of Public Health and Environmental Science, Ishikawa, Japan; ^21^​ National Institute of Infectious Diseases, Tokyo, Japan

**Keywords:** Shiga toxin-producing *Escherichia coli *O121:H19, comparative genomics, phylogenetic analysis, population structure, bacteriophage, plasmid

## Abstract

Shiga toxin (Stx)-producing *Escherichia coli* (STEC) are foodborne pathogens causing serious diseases, such as haemorrhagic colitis and haemolytic uraemic syndrome. Although O157:H7 STEC strains have been the most prevalent, incidences of STEC infections by several other serotypes have recently increased. O121:H19 STEC is one of these major non-O157 STECs, but systematic whole genome sequence (WGS) analyses have not yet been conducted on this STEC. Here, we performed a global WGS analysis of 638 O121:H19 strains, including 143 sequenced in this study, and a detailed comparison of 11 complete genomes, including four obtained in this study. By serotype-wide WGS analysis, we found that O121:H19 strains were divided into four lineages, including major and second major lineages (named L1 and L3, respectively), and that the locus of enterocyte effacement (LEE) encoding a type III secretion system (T3SS) was acquired by the common ancestor of O121:H19. Analyses of 11 complete genomes belonging to L1 or L3 revealed remarkable interlineage differences in the prophage pool and prophage-encoded T3SS effector repertoire, independent acquisition of virulence plasmids by the two lineages, and high conservation in the prophage repertoire, including that for Stx2a phages in lineage L1. Further sequence determination of complete Stx2a phage genomes of 49 strains confirmed that Stx2a phages in lineage L1 are highly conserved short-tailed phages, while those in lineage L3 are long-tailed lambda-like phages with notable genomic diversity, suggesting that an Stx2a phage was acquired by the common ancestor of L1 and has been stably maintained. Consistent with these genomic features of Stx2a phages, most lineage L1 strains produced much higher levels of Stx2a than lineage L3 strains. Altogether, this study provides a global phylogenetic overview of O121:H19 STEC and shows the interlineage genomic differences and the highly conserved genomic features of the major lineage within this serotype of STEC.

## Data Summary

The raw read sequences and complete genome sequences generated for this study have been deposited in GenBank/EMBL/DDBJ under the BioProject accession number PRJDB8147 (https://www.ncbi.nlm.nih.gov/bioproject).

**Impact Statement**

Shiga toxin (Stx)-producing *Escherichia coli* (STEC) are important foodborne pathogens that cause not only mild enteritis but also severe haemorrhagic colitis and life-threatening haemolytic uraemic syndrome. Among STEC strains of various serotypes, O157 STEC is the most predominant worldwide, but infections by several non-O157 STECs have recently increased, including O121:H19 STEC. However, due to the lack of systematic whole genome sequence (WGS) analyses, the population structure and genomic diversity of O121:H19 STEC are unknown. Here, we produced genome sequences of 143 strains, including four complete genomes, to expand the genomic information resource of O121:H19 and performed a global WGS analysis, a detailed comparison of complete genomes, and analyses of the Stx2 phage genomes of selected strains and their Stx2 production levels. Through these analyses, we show that O121:H19 comprises four lineages, including the major and second major lineages circulating worldwide. The evolutionary history of the acquisition of major virulence factor-encoding genetic elements, a notable difference in the prophage pool between the major and second major lineages, and a high conservation of prophages in the major lineage were also revealed. Thus, this study revealed, for the first time, a global population structure and notable interlineage differences within this important but understudied STEC.

## Introduction

Shiga toxin (Stx)-producing *Escherichia coli* (STEC) are foodborne pathogens that cause a range of diseases, from mild enteritis to severe haemorrhagic enteritis, and sometimes life-threatening complications such as haemolytic uraemic syndrome (HUS) [1]. While there are STEC strains with various serotypes, those with O157:H7 serotype (O157 STEC) have been the most predominant worldwide. However, STEC infections by STEC strains with non-O157:H7 serotypes (non-O157 STEC) have increased in recent years. In the United States and Europe, the total number of reported non-O157 STEC infections has exceeded that of O157 since 2013 and 2007, respectively, and many outbreaks of non-O157 STEC infections have been reported [2, 3].

The major virulence factors of typical STECs are Stxs encoded by prophages and the type III secretion system (T3SS) encoded by the locus of enterocyte effacement (LEE). Stxs are classified into two subtypes, Stx1 and Stx2, each of which is further classified into several variants [4, 5]. Although several T3SS-secreted effectors are encoded by the LEE, many additional effectors are encoded by prophages [6–8]. There are also many additional potential virulence factors encoded by prophages, integrative elements, or virulence plasmids, but their involvement in STEC pathogenicity has not yet been fully clarified. Interestingly, previous studies have shown that STEC strains with different serotypes have emerged independently by acquiring a similar set of virulence genes via horizontal gene transfer mediated by these mobile genetic elements (MGEs) [8–10]. However, even within the same serotype, there are marked variations in the repertoire of virulence factor-encoding MGEs, including the prophages that encode *stx* genes (Stx phages) [11–15]. Lineage-dependent variation in clinical severity, such as the frequency of HUS, has also been observed in O157 and O26 STEC, in which highly virulent clones or clades have been identified [16–21].

O121:H19 STEC is one of the six major non-O157 STECs along with the O26, O103, O111, O145, and O45 STECs [22]. Similar to other major STECs, it causes haemorrhagic enteritis sometimes associated with HUS [23]. A large flour-associated outbreak by O121:H19 STEC occurred recently in the USA [24]. In Japan, O121:H19 STEC accounts for 1.9–3.0 % of the annually reported STEC infections and represents the fourth most common serotype after O157, O26, and O103 [25]. In contrast, there are fewer reports of O121:H19 infection in Europe [3, 26]. Although whole genome sequence (WGS) analyses have been reported for limited numbers of strains and outbreaks [24, 27, 28] and the main sequence type (ST) of O121:H19 based on the Achtman’s scheme of multi-locus sequence typing (MLST) [29] has been reported to be ST655 [27, 30], no systematic WGS-based analysis of O121:H19 has been conducted. Therefore, its global population structure and genomic diversity are unknown.

In this study, to reveal the general genomic features, global population structure, and genomic diversity of O121:H19 STEC, we performed a WGS analysis of 638 O121:H19 strains, including 143 strains sequenced in this study, and a detailed comparison of 11 complete genomes, including four complete genomes obtained in this study. The results of the analyses of the Stx2 phage genomes and Stx2 production levels of selected O121:H19 strains are also described.

## Methods

### Bacterial strains

All 143 O121:H19 strains sequenced in this study were human isolates. Of these, 138 were isolated in various regions of Japan between 1997 and 2016. The other five strains were isolated in Belgium between 1996 and 2010. To collect publicly available genome sequences of O121:H19 strains, read or assembled sequence data were downloaded from the NCBI and EnteroBase [31] (https://enterobase.warwick.ac.uk/) databases (final access: 10 October 2019). After confirming their serotypes as previously described [32], low-quality sequences were excluded (coverage depth; <20× or contamination determined by CheckM [33]; >2 %). ST determination was performed by a read mapping-based or blastn-based strategy as previously described [34]. To analyse the phylogenetic relationship between ST655 and its close variants, we further collected genome sequences of the single locus variants (SLVs) and double locus variants (DLVs) of ST655 from the NCBI and EnteroBase databases (final access: 24 November 2020). The SLVs and DLVs differ from the allelic profile of ST655 at one locus (SLVs) or two loci (DLVs) among the seven loci analysed (*adk*, *fumC*, *gyrB*, *icd*, *mdh*, *purA*, and *recA*). Zhou *et al.* showed that *E. coli* strains belonging to an ST and its SLVs and DLVs are phylogenetically close to each other by a core gene-based MLST analysis (cgMLST) [31]. Serotypes of strains were determined by SerotypeFinder [35]. The final set analysed in this study included 638 O121:H19 strains (ST655 or its SLVs) and 42 non-O121:H19 strains (all were DLVs of ST655), as listed in Tables S1 and S2 (available in the online version of this article) respectively.

### Genome sequencing, assembly, and annotation

Purification of genomic DNA, preparation of sequencing libraries, Illumina sequencing, and sequence assembly were performed as previously described [32], except for the sequencing of strain 51104. The sizes of assembled genomes, sequence coverages, and yielded scaffolds ranged from 5015 kb to 5467 kb (average: 5271 kb), from 37× to 187× (average: 74×), and from 171 to 569 (average: 278), respectively. Strain 51104 was sequenced using Roche 454 GS FLX to generate 487397 reads (average read length: 242 bp), and the reads obtained were assembled by Roche Newbler to generate 228 contigs. To fill the gaps in the assemblies, a plasmid-based shotgun library (insert size: approximately 3 kb) and a fosmid library (insert size: approximately 40 kb) were prepared and end-sequenced by ABI 3730xl (7680 clones and 3840 clones, respectively) to scaffold the contigs. Then, gaps were closed by direct sequencing of gap-covering fosmid clones or gap-spanning PCR products using ABI 3130xl or ABI 3730. Finally, sequence errors in the closed chromosome and plasmid sequences were corrected by mapping 65 bp read sequences obtained by Illumina GAIIx using the MAQ programme [36].

To determine the complete sequences of strains E15042, SE14002 and CEC14159, their genomes were additionally sequenced using MinION with R9.4.1 flow cells (Nanopore) for 48 h (E15042) or 67 h (SE14002 and CEC14159). Read data in fastq format were generated using MinKnow v1.14.1 and Albacore v2.3.1 (E15042) or MinKnow v1.15.4 and qcat v1.0.1 (SE14002 and CEC14159). Nanopore reads were trimmed and filtered using the following programme and parameters: trimming by porechop (v0.2.2) [37] and filtering (E15042) over 2 kb at a quality score (Q score) of >15 by NanoFilt (v2.3.0) [38] or filtering (SE14002 and CEC14159) over 2 kb at a Q score of >10 by NanoFilt (v2.3.0) with the option of trimming 100 bp from the start of reads. The filtered nanopore reads were assembled along with the trimmed Illumina reads of each strain using Unicycler v0.4.6 (E15042) or v0.4.7 (SE14002 and CEC14159) [39]. The complete genome sequences determined in this study were annotated using DFAST [40], followed by manual curation. Prophages, integrative elements and ISs were identified as previously described [32]. GenomeMatcher (v2.3) [41] was used for genome sequence comparison and to display the results.

The complete genome sequences of strains 51104, E15042, SE14002 and CEC14159 and the short-read sequences of 139 O121:H19 strains obtained in this study have been deposited in DDBJ/EMBL/GenBank under BioProject accession numbers starting from PRJDB8147 (see Table S1 for each accession number).

### SNP detection and phylogenetic analyses

Phylogenetic analyses of two strain sets, one including an ST655 strain and 13 strains of single locus variants (SLVs) or double locus variants (DLVs) of ST655 (*n*=14) and the other including all O121:H19 strains (*n*=638), were performed using O121:H19 strain 51104 (ST655) as a reference. For the former set, eight strains were selected from each of the eight SLVs of ST655. The remaining five strains were selected from the DLVs of ST655; one was representative of ST1686, and four ST2952 strains were representatives of each of the four serotypes found in this ST. These 14 strains are indicated in Tables S1 and S2.

SNP sites on prophage/integrative element/IS-free and recombination-free chromosome backbone sequences conserved in all genomes analysed (referred to as ‘core genome’) were identified using Gubbins [42] and MUMmer [43] and used for the construction of a maximum likelihood (ML) tree with RAxML [44] as previously described [32]. In the entire O121:H19 phylogenetic tree construction, strains were deduplicated if the recombination-free core sequences were identical. Clustering analysis was performed using the hierBAPS programme with the parameters L=2 and maxK=4 [45]. ML trees were displayed using iTOL [46] or FigTree v.1.4.4 (http://tree.bio.ed.ac.uk/software/figtree/).

### Analyses of *stx*, *eae* and *hlyA* genes, plasmid replicons, and T3SS effector repertoires

The subtypes of *stx* and *eae* were determined by blastn as previously described [34]. The presence of *hlyA* was also determined by blastn search (>90 % identity and >90 % coverage) using the *hlyA* sequence of strain 51104 (EC51104_p1-02) as a query sequence. Plasmid replicons were identified using PlasmidFinder v.2.0.1 [47] and the PlasmidFinder Database (Database version: 2021-01-13) with default parameters. T3SS effector repertoires in the 11 complete genomes were analysed by BLASTX search as previously described [48].

### Analysis of Stx2a phages

In the 49 O121:H19 strains that were available in our laboratory, the integration of Stx2a phages into the *argW* or *yecE* locus was analysed by long PCR, which was followed by Illumina sequencing and assembly of PCR products as previously described [34]. In brief, the segments of Stx2a prophage regions at *argW* and *yecE* were amplified using four or two pairs of primers, respectively, as illustrated in Fig. S1. Sequences of the primers used are also shown in Fig. S1. Gene annotation of Stx2a phage genomes was carried out with DFAST. All Stx2a phage genome sequences determined in this study have been deposited in the DDBJ/EMBL/GenBank databases under the accession numbers listed in Table S3.

The phylogeny of the 49 O121:H19 strains whose Stx2a phage genomes were sequenced were reconstructed using their genome sequences along with those of ten strains whose complete genome sequences were determined (referred to as ‘completely sequenced strains’) as described above. To compare the Stx2a phage genomes integrated in *argW* (47 were individually sequenced as described above, and ten were extracted from the complete genomes), we first identified IS elements in the Stx2a phage genomes using ISFinder [49]. Then, each IS-free phage genome was aligned with the IS-free Stx2a phage genome of strain 4151 using NUCmer v3.1 [43] to detect mismatches, insertions/deletions (indels), and gaps.

### Determination of Stx2 production levels

Overnight cultures were inoculated in 2 ml of lysogeny broth at an OD_600_ of 0.1 and grown to mid-log phase at 37 °C with shaking. Then, mitomycin C (MMC; Kyowa Kirin) was added to the cultures at a final concentration of 0.5 µg ml^−1^. After 6 h of incubation, cell lysates were prepared, and the Stx2 concentration in each lysate was determined using sandwich ELISA as previously described [50]. MMC concentration and sampling time were optimized based on the results of exploratory analyses of four strains (see Fig. S2 for details).

### Temporal analysis

By excluding 63 genomes lacking temporal information, 575 O121:H19 genomes and an outlier (that of O64:H19 strain NCTC9064) were selected, and an ML tree was constructed using 6976 recombination-free SNP sites in their core genome sequence (3321265 bp in length) by the same method as described above. Based on this information, the strain set was down-sized to 228 genomes using Treemmer v.0.3 with RTL (relative tree length) option 0.95 [51]. The 228 strains are indicated in Tables S1 and S2. Using the 6923 recombination-free SNP sites in the core genome of these 228 genomes (3437152 bp), an ML tree was generated again, and the temporal signal in the tree was examined using TempEst [52] by assessing the linear relationship between the root-to-tip distance and the year of isolation. The GTR substitution model with the strict clock and constant population size model was selected as the best-fit model by assessing the Bays factor. Subsequent temporal analysis was performed using BEAST v.1.8.4 [53] as previously described [32]. The result was summarized as a maximum clade credibility tree using TreeAnnotator in BEAST and visualized with FigTree v.1.4.4.

## Results and discussion

### Strain set

We sequenced 143 O121:H19 strains (138 Japanese and five Belgian strains) in this study and collected publicly available genome sequences of O121:H19 strains and those belonging to SLVs and DLVs of ST655, the major ST of O121:H19. After excluding low-quality sequences, the final set included the genome sequences of 680 strains from various geographic regions (see Tables S1 and S2 for details).

The serotypes of strains belonging to nine SLVs of ST655 (ST5772/6689/7245/1869/5536/8892/6000/800/7250) were all O121:H19 (Table S1; note that genome sequences of ST1869 were not available). Among the four DLVs of ST655, genome sequences were available for two DLVs (ST1686 and ST2952). The serotype of the ST1686 strains (*n*=4) was O8:H26, and those of the ST2952 strains (*n*=38) were also non-O121:H19 (O20:H9, O57:H19, O64:H19, or O168:H9; Table S2).

### Phylogenetic relationship of the strains belonging to ST655 and its SLVs and DLVs

**Fig. 1.**
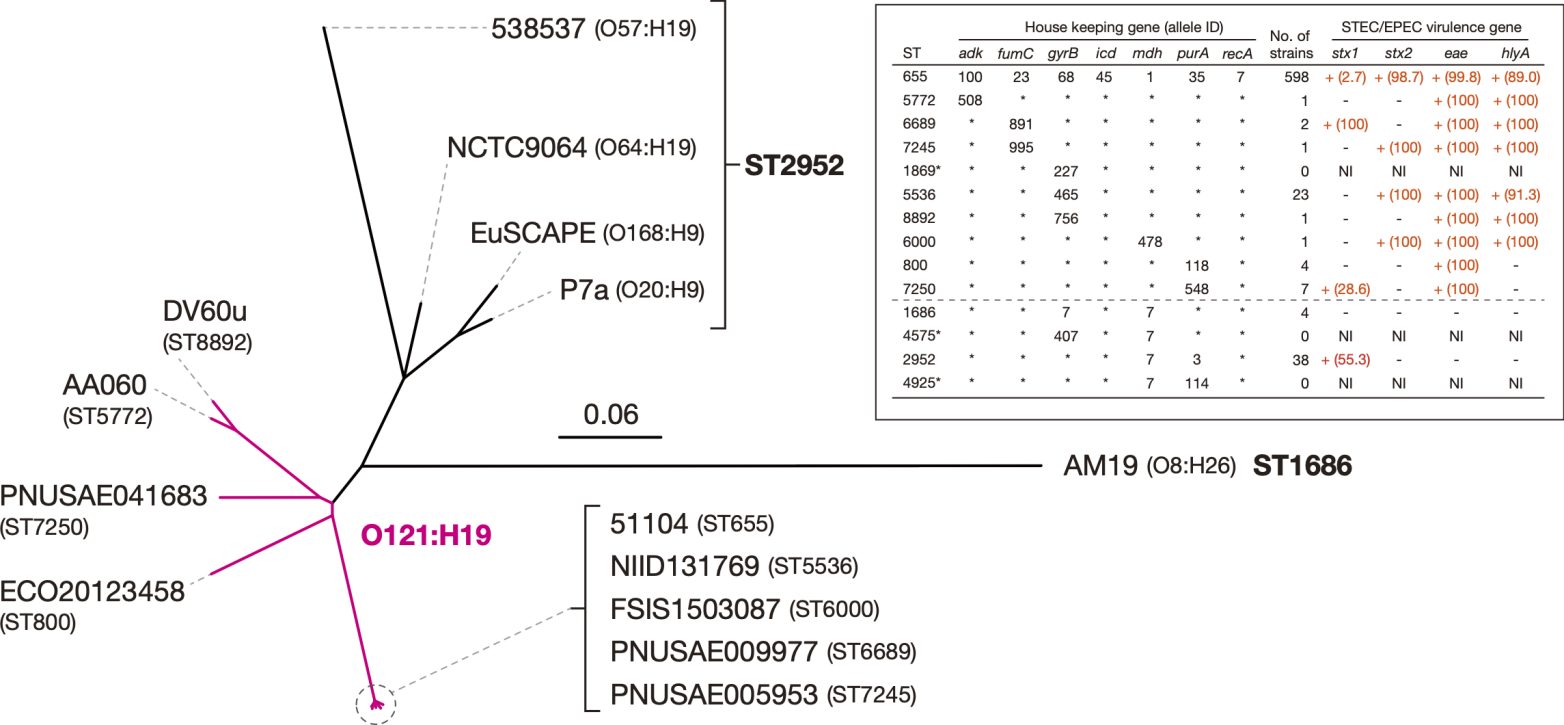
Phylogenetic relationships between O121:H19 strains and their close relatives. An unrooted ML tree was constructed based on the recombination-free SNPs (4056 sites) identified on the chromosomal backbone sequence (3424039 bp). Strain names are displayed on each tip with their STs or serotypes indicated in parentheses. The O121:H19 branches are shown in magenta. Information on allele IDs for MLST, the distribution of *stx1*, *stx2*, *eae*, and *hlyA* genes (major virulence genes of STEC/EPEC) in ST655, and the SLVs and DLVs of ST655 are presented in the inset. Asterisks indicate STs with no available genome sequence information. Regarding the distribution of virulence genes of each ST, when the gene was detected in at least one strain in an ST, the ST was regarded as positive. The proportions (%) of positive strains in each ST were indicated in parentheses. NI, no information. Bar, the mean number of nucleotide substitutions per site.

To reveal the phylogenetic relationship between ST655 and its SLVs and DLVs, we first constructed a WGS-based phylogenetic tree of representative strains that were selected from each ST and/or serotype (Fig. 1). All SLVs formed a cluster with ST655, and four of the eight SLVs (ST5536, ST6000, ST6689, and ST7245) were very closely related to ST655, but the other four SLVs were distantly related to ST655 and notably diverged from each other. Four strains that belonged to ST2952 (one of the two DLVs) but had different serotypes formed a cluster distinct from that of ST655 and its SLVs. The strain belonging to ST1686 (another DLV) formed a distinct branch in the tree.

We next analysed the distribution of four marker genes for typical STECs (*stx1*, *stx2, eae*, and *hlyA*) in the 680 strains used in this study. As shown in the summary table superimposed in Fig. 1 (note that ‘positive’ indicates that the gene was detected in at least one strain in each ST), not only *stx1* and *stx2* but also *eae* (the marker of the LEE) and *hlyA* (the marker of virulence plasmids of typical STECs) were present in ST655. In the SLVs, while the distribution of *stx* genes was variable, *eae* was detected in all SLVs, and *hlyA* was also detected in six SLVs other than ST800 and ST7250. In contrast, *eae* and *hlyA* were not detected in any strains belonging to the two DLVs (ST2952 and ST1686), while *stx1* was detected in ST2952 (21 out of 38 strains). These findings suggest that the common ancestor of O121:H19 acquired the LEE and the virulence plasmid and then separated into several sublineages within O121:H19. It seems most likely that the virulence plasmid has been lost in the ST800 and ST7250 sublineages, but we cannot exclude the possibility that it was acquired independently by the ST655/5536/6000/6689/7245 and ST5772/8892 sublineages.

### Phylogenetic overview of O121:H19

We analysed the WGSs of a total of 638 O121:H19 strains isolated in 11 countries, of which 143 were sequenced in this study (Tables 1 and S1). Among the 638 strains, 232 were identical to one or more strains in the strain set at the core genome level and formed 36 subsets of strains with identical core genome sequences (Table S1). These 36 subsets of strains included those associated with three outbreaks in the USA [24], Canada [54] and Japan [28], while epidemiological links for others are unknown. To reduce strain redundancy, one representative strain was selected from each subset and included in the following analyses. Therefore, our final strain set included a total of 442 strains.

**Table 1.** The O121:H19 strain set analysed in this study

Country	Sources						*stx* genotypes			
	Human	Animals	Foods	Environment	No information	Total	*stx1a*	*stx1a*/ *stx2a*	*stx2a*	Negative
Japan	196	2	0	1	2	201	1	5	195	0
United States	242	21	55	8	34	360	10	4	338	8
Canada	38	0	15	1	3	57	0	0	57	0
European countries^*^	8	0	1	0	3	12	0	0	12	0
Other countries^†^	1	1	0	0	1	3	0	0	0	3
No information	0	0	0	0	5	5	0	0	4	1
Total	485	24	71	10	48	638	11	9	606	12

*Belgium (*n*=5), Finland (*n*=3), Denmark (*n*=2), Italy (*n*=1), France (*n*=1)

†Korea (*n*=1), New Zealand (*n*=1), South Africa (*n*=1)

**Fig. 2.**
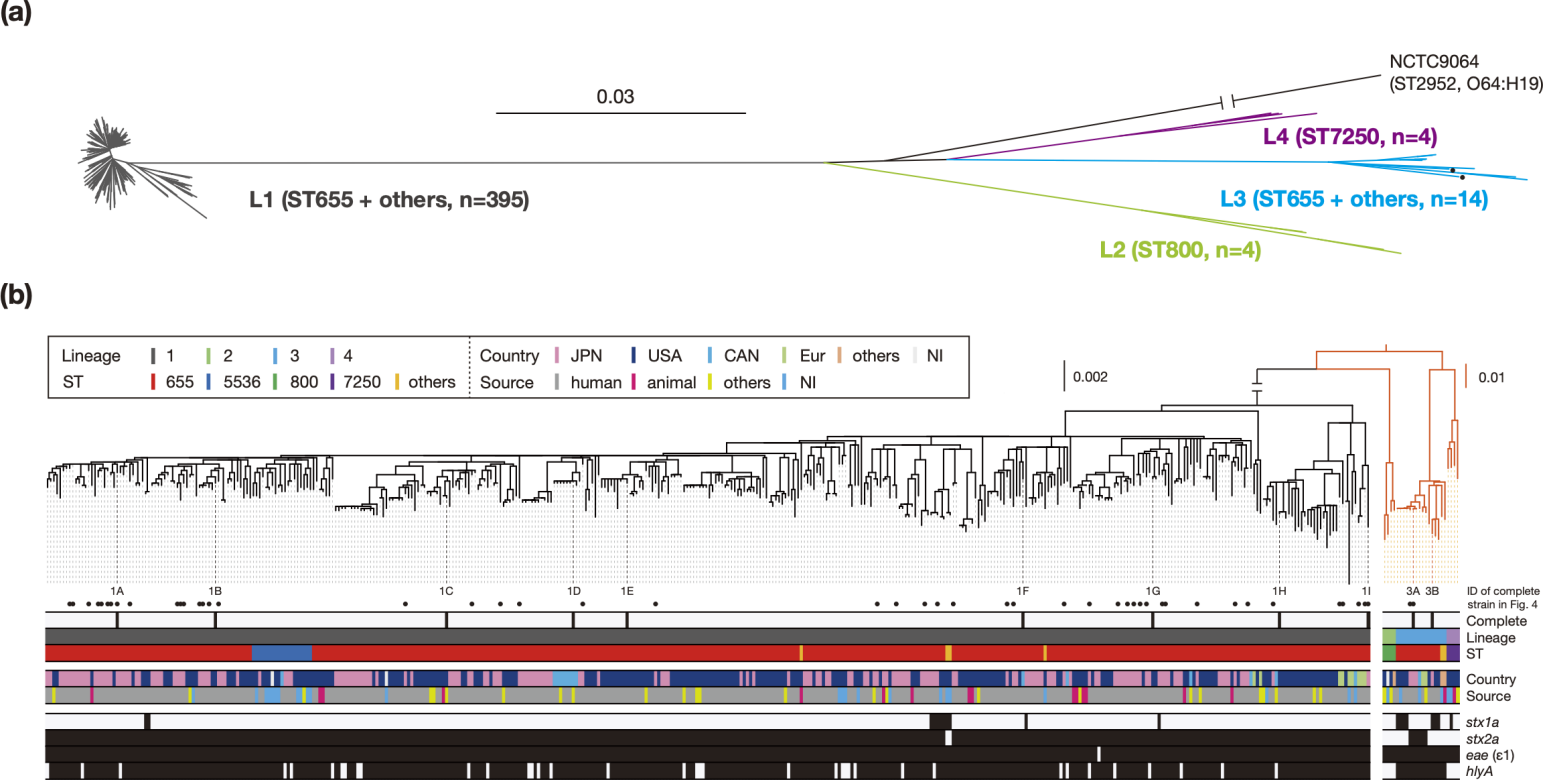
Phylogenetic relationship of the 442 O121:H19 strains. ML trees were reconstructed based on the recombination-free SNPs (7371 sites) identified on the chromosomal backbone sequence (3312074 bp) with an O64:H19 strain (NCTC9064) belonging to ST2952 (DLV of ST655) as an outgroup. In panel (**a**), four lineages identified by hierBAPS (L1-L4) are indicated with the names of the main ST and the number of strains in parentheses. L1 includes strains belonging to four STs (ST5536, ST6000, ST6689, and ST7245). L3 includes ST5772 and ST8892 (one strain each), which are indicated by dots. In panel (**b**), strain information was mapped on an ML tree. Branches for non-L1 lineages are indicated in orange with a genetic distance scale different from that for L1. Completely sequenced strains (*n*=11) are indicated (1A, 51104; 1B, 2014C-3599; 1C, RM8352; 1D, 16–9255; 1E, 2015C-3107; 1F, FWSEC0006; 1G, 2014C-3655; 1H, 2014C-4423; 1I, E15042; 3A, SE14002; and 3B, CEC14159). The presence or absence of the *stx1*, *stx2*, *eae*, and *hlyA* genes is indicated by a filled or open box, respectively. JPN: Japan, USA: United States of America, CAN: Canada, Eur: European countries, NI: No information. Bar, the mean number of nucleotide substitutions per site.

Almost all of the strains (409 out of 442 strains) belonged to ST655, while the others belonged to SLVs of ST655: ST800 (*n*=4), ST5536 (*n*=19), ST7250 (*n*=4), ST6689 (*n*=2), and ST5772, ST6000, ST7245 and ST8892 (*n*=1 in each). By core genome-based phylogenetic analysis and hierBAPS-based clustering, the 442 O121:H19 strains were divided into four lineages named L1-L4 (Fig. 2a). The major lineage (L1) comprised strains belonging to ST655 (*n*=395) and several non-ST655 strains (*n*=23). Unexpectedly, the second major lineage (L3) also included ST655 strains (*n*=14), along with one ST5772 and one ST8892 strain. Two minor lineages comprised ST800 and ST7250 strains, respectively. The presence of ST655 in both L1 and L3 and the phylogenetic relationship of the four O121:H19 lineages relative to ST2952 strains (DLV of ST655), which was used as an outgroup of phylogenetic analysis, suggested that it is most likely that ST655 is ancestral to the entire O121:H19 lineage.

Japanese and USA isolates, which represented 39 and 54 % of the entire strain set, respectively, were distributed throughout lineage L1. Canadian (*n*=16) and European (*n*=11) isolates were also included in lineage L1, indicating the global circulation of this lineage. Most L1 strains (*n*=354) were human isolates (Table 1, Fig. 2b). Strains isolated from animals, food, and the environment (the latter two were labelled ‘others’ in Fig. 2b) were distributed between human strains. Thus, it seems that there is no obvious strain bias derived from isolation sources in this strain set.

Analysis of the distribution of major virulence-related genes revealed that *stx2a* was harboured by almost all L1 strains (416 out of 418 strains; Fig. 2b) and a portion of the L3 lineage (six out of 16 strains). This finding raised the possibility that the Stx2a phage was acquired by a common ancestor of L1 and has been stably maintained in the lineage. The *stx1a* gene was present in a small number of strains (*n*=20) belonging to three lineages (L1, L3, and L4) with a sporadic distribution pattern. All O121:H19 strains except for one strain contained the *eae* gene of subtype ε1 (Fig. 2b, Table S1), suggesting that the LEE has been stably maintained in the entire O121:H19 lineage. In contrast, the distribution of the *hlyA* gene was limited to L1 and L3, raising two possibilities: (i) the virulence plasmid was acquired by the common ancestor of O121:H19 and then lost in the ST800 and ST7250 strains or (ii) it was independently acquired by the ancestors of L1 and L3.

### Analysis of complete O121:H19 genomes

#### General features

Complete genome sequences for seven O121:H19 strains were publicly available [54–57], but all were found to belong to lineage L1 (Fig. 2b). To capture a more complete view of the genomic features of O121:H19, we selected two strains from two understudied sublineages of L1 (strains 51104 and E15042 referred to as 1A and 1I, respectively) and two strains from the second major lineage L3 (strains SE14002 and CEC14159 referred to as 3A and 3B, respectively) and determined their complete genome sequences. In this manuscript, the seven previously sequenced L1 strains (strains 2014C-3599, RM8352, 16–9255, 2015C-3107, FWSEC0006, 2014C-3655, and 2014C-4423) were referred to as 1B, 1C, 1D, 1E, 1F, 1G, and 1H, respectively (Fig. 2b, Table 2). The STs of all strains were ST655 in the Achtman’s typing scheme. Based on the the EcMLST’s scheme using seven allele sequences [58], they were assigned to ST-182 except that 1A was found to be its SLV.

**Table 2.** General genomic features of the eleven completely sequenced O121:H19 STEC strains

Sequence Type (ST)	655	655	655	655	655	655	655	655	655	655	655
Lineage*	1	1	1	1	1	1	1	1	1	3	3
Strain	51104	2014C- 3599†	RM8352‡	16–9255‡	2015C- 3107†	FWSEC0006‡	2014C- 3655†	2014C- 4423†	E15042	SE14002	CEC14159
Strain ID in this paper	1A	1B	1C	1D	1E	1F	1G	1H	1I	3A	3B
Accession No.	AP024471- 2	CP027435- 6	CP028110- 1	CP022407- 8	CP027317- 8	CP031910-1	CP027350- 1	CP027454- 6	AP024478- 9	AP024473- 4	AP024475- 7
Reference	This study	Patel PN, *et al*. [55]	Parker CT, *et al*. [56]	Robertson J, *et al*. [54]	Patel PN, *et al*. [55]	Tyson S, *et al*. [57]	Patel PN, *et al*. [55]	Patel PN, *et al*. [55]	This study	This study	This study
Chromosome (kb)	5391	5400	5391	5398	5388	5399	5443	5339	5365	5236	5221
CDSs	5205	5228	5249	5249	5229	5226	5291	5199	5194	5000	4993
rRNA operons	7	7	7	7	7	7	7	7	7	7	7
tRNAs	99	102	102	102	102	102	102	101	101	102	105
Prophages	15	15	15	15	15	15	16	15	14	13	14
Integrative elements	5	5	5	5	5	5	5	5	5	6	5
Plasmid (kb)	82	84	83	82	82	81	97§	80/73	81	88	89/88
CDSs (plasmid total)	84	88	89	80	83	78	125	175	82	89	181
Total genome size (kb)	5473	5484	5474	5480	5470	5480	5540	5492	5446	5324	5398

*Determined by hierBAPS.

†Annotated using DFAST in this study.

‡Re-annotated using DFAST in this study.

§Plasmid is not closed.

As summarized in Table 2, the chromosomes of the 11 strains were 5221–5443 kb in size and contained various numbers of prophages (13-15) and integrative elements (five or six). These strains carried one or two large plasmids (81–97 kb in size; note that the 97 kb plasmid of strain 2C was not closed). The chromosome backbones of the 11 strains were well conserved and exhibited overall genomic synteny, except for inversions found in several strains (Fig. S3). For IS elements, while we analysed only four strains sequenced in this study (two L1 strains and two L3 strains), we found marked differences in the IS repertoire between the two lineages; among the 40 types of IS elements (12 families) found in the four strains, nine were found only in L1 strains, and 11 were found only in L2 strains (Table S4). In contrast, the IS repertoire exhibited high intralineage conservation, and the copy numbers of each IS element were also well conserved (same or within two copy differences except for three IS elements) within each lineage. These interlineage differences and intralineage conservation of IS elements appear to reflect the independent evolution of each lineage.

#### Variation in prophages

**Fig. 3.**
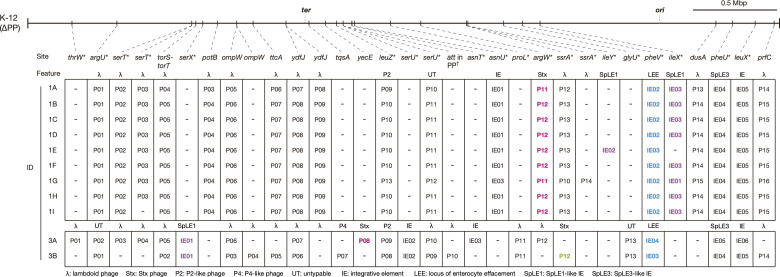
Conservation and variation of the prophages and integrative elements in the 11 complete O121:H19 genomes. The chromosomal integration sites of prophages and integrative elements (IEs) identified in the 11 complete genomes are shown on the prophage-removed chromosome backbone (K-12 ∆PP) of K-12 MG1655. Insertion sites are indicated by gene names or intergenic regions with tRNA and tmRNA genes marked by asterisks. ‘*att* in PP’ indicated by a dagger (†) indicates an *attB* site within the prophage genome integrated into the *serU* gene (see Fig. S3 for more details). Prophages encoding *stx2* and *stx1* are indicated by red and green, respectively. The IEs corresponding to the LEE and SpLE1 are indicated by blue and magenta, respectively.

We identified a total of 23 prophage integration sites, including four sites where two prophages were inserted in tandem (Fig. 3). Among the nine L1 strains, a total of 16 sites were identified, and 14 of them were shared by all strains, including the Stx2 phage integration site (*argW*). The only exceptions were the *serT* and *ssrA* loci, each specific to strains 2A and 2C. In contrast, in the two L3 strains, while a total of 20 sites were identified, only eight sites were shared by the two strains, and six of them (*argU*, *torS*/*torT*, *ompW*, *ydfJ*, *leuZ*, and *serU*) were also shared by all L1 strains. The remaining 12 sites, including the Stx2 phage integration sites, were specific to each strain. It should be noted that one of the integration sites specific to strain 3B was found in the genome of a prophage integrated in *serU* (Fig. S4). This type of phage integration (prophage integration into prophages) was recently found in several STEC strains of various serotypes [34].

We compared the sequences of prophage genomes found in each strain by dot plot analysis (identity threshold: >99 %) using those of strain 1A (21 prophages) as references (Fig. S3). Among the L1 strains, the prophages integrated in the same site exhibited a high sequence similarity except for small deletions detected in several cases (Fig. S3a). This analysis also revealed that all of the large chromosome inversions observed between the L1 strains occurred between the prophages (between the prophages at *ydfJ* and *ttcA* or between those at *ydfJ* and *dusA*). The prophage of L3 strains at the six sites was shared by all L1 and L3 strains and had genome sequences different from the counterparts of strain A1, except for those at *ompW* and *leuZ*, although some sequences were partially conserved (Fig. S3b). The chromosome inversion found in the two L3 strains also occurred between the prophages at *ydfJ* and *ttcA*. Among the prophages found at the eight sites common to both strains, those at six sites (*argU*, *torS*/*torT*, *ompW*, *leuZ*, *proL*, and *glyU*) had well conserved sequences. However, those at the remaining two sites (*ydfJ* and *serU*) shared no homologous regions with each counterpart other than short homologous sequences (Fig. S3c).

These findings suggest that (i) there is a marked difference in prophage repertoires between L1 and L3 with only a small number of shared prophages, (ii) most of the prophages found in L1 strains were acquired by the common ancestor of this lineage and have stably been maintained, and (iii) there is a notable difference in prophage pool within L3.

#### Variation in integrative elements (IEs)

We identified nine integration sites for IEs in the 11 strains. At three sites (*pheV*, *pheU*, and *leuX*), IEs were found in all strains, and these IEs had well-conserved genome sequences except for that found at *pheV* in strain 3A (Fig. S3). The IEs at *pheV* and *pheU* correspond to the LEE and the SpLE3-like element [59], respectively, the latter of which also encodes T3SS effectors (*nleB*, *nleE*, and *espL*). The high conservation of LEE sequences is consistent with the aforementioned results of *eae* subtype analysis, which suggested that the LEE was acquired by the common ancestor of O121:H19, but the LEE of strain 3A contained a long accessary region unique to this strain (Fig. S5a).

Of the other six sites, two (*serX* and *serU*) were L3-specific IE integration sites, and the same IEs were present in the two L3 strains. The IEs at *serX* were SpLE1-like elements encoding the tellurium-resistance (*ter*) and urease (*ure*) operons and the *iee* gene [60] (Fig. S5b). Among the four L1-specific IE integration sites, the same 9.5 kb IE was found at *asnU* in all L1 strains. At the *ileX* locus, nearly identical SpLE1-like elements were present in eight L1 strains, but this element was translocated to the *ileY* locus with a genome rearrangement in strain 1E (Fig. 3 and Fig. S5b; note that the *ileX* and *ileY* loci share the same *attB* sequence). The SpLE1-like elements of lineages L1 and L3 differed in not only insertion site but also size and gene organization, suggesting that they were acquired independently by the ancestors of each lineage (Fig. S5b).

#### Variation in T3SS effectors encoded by prophages and IEs

In the 11 strains, we identified many genes encoding T3SS effectors belonging to 26 effector families (33–48 copies per genome) (Table S5). The effector repertoires of the nine L1 strains were very similar; they encoded the same 25 effector families (45–48 copies per genome). In contrast, 23 families (43 copies) and 23 families (33 copies) were encoded by the two L3 strains 3A and 3B, respectively. Of the 25 families found in L1 strains, *espW* and *tccp* were absent in the two L3 strains, and *espN* and *tccp2* were absent in 3A and 3B, respectively. The *espV* and *nleD* genes were found only in strain 3A among the 11 strains, although the *espV* gene was degraded. Thus, there were slight differences in the effector repertoire between L1 and L3 and between L3 strains. Since most non-LEE effectors were encoded by prophages except for the aforementioned three genes encoded by SpLE3, this result reflected the conservation and variation of prophages between the 11 strains.

#### Variations in plasmids

Virulence plasmids were highly conserved in sequence and gene organization within lineages L1 and L3, respectively, except for structural variations due to IS-related deletions and inversions (Fig. S6a). However, there were marked differences between the virulence plasmids of the L1 and L3 strains, and only the regions encoding the *ehx* and *ecf* operons were shared (Fig. S6b), suggesting that the two lineages independently acquired virulence plasmids with distinct backbones. Among the virulence plasmids of other serotypes of STEC, that of L1 is similar to pO26 and pO145 and that of L3 is similar to pO157, but to a lesser extent (Fig. S6c).

Additional plasmids that did not encode genes apparently related to virulence or antimicrobial resistance (referred to as nonvirulence plasmids) were present in two strains, 1H and 3B (Table 2). They were different plasmids, but both carried a set of conjugation-related genes. That of strain 3B additionally encoded an operon for the synthesis of type IV pilli (Fig. S6d) and was highly similar to a plasmid of *Salmonella* species [61] (>95 % nucleotide sequence identity in nearly entire genomes; Fig. S6e), suggesting interspecific transmission of this plasmid.

These findings suggested the high conservation of virulence plasmids and frequent gain and loss of other plasmids in the entire O121:H19 lineage, as observed in the O145:H28 STEC lineage [32]. This notion was confirmed by an additional analysis of the repertoires of plasmid replicons using PlasmidFinder [47] (Fig. S7), which detected 25 types of replicons in the 422 strains analysed in this study. The virulence plasmids of the L1 type (containing the *IncFIB* and *IncB*/*C*/*K*/*Z* replicons) and that of the L3 type (*IncFIB* and *IncFII*) were well conserved in each lineage. In contrast, the distributions of other plasmid replicons were nearly strain specific, and even the most frequently detected replicon, *IncI2* (Delta), was found in only 10.7 % of the 422 strains.

#### Variation in Stx2 production level and the dynamics of Stx2a phages in O121:H19

**Fig. 4.**
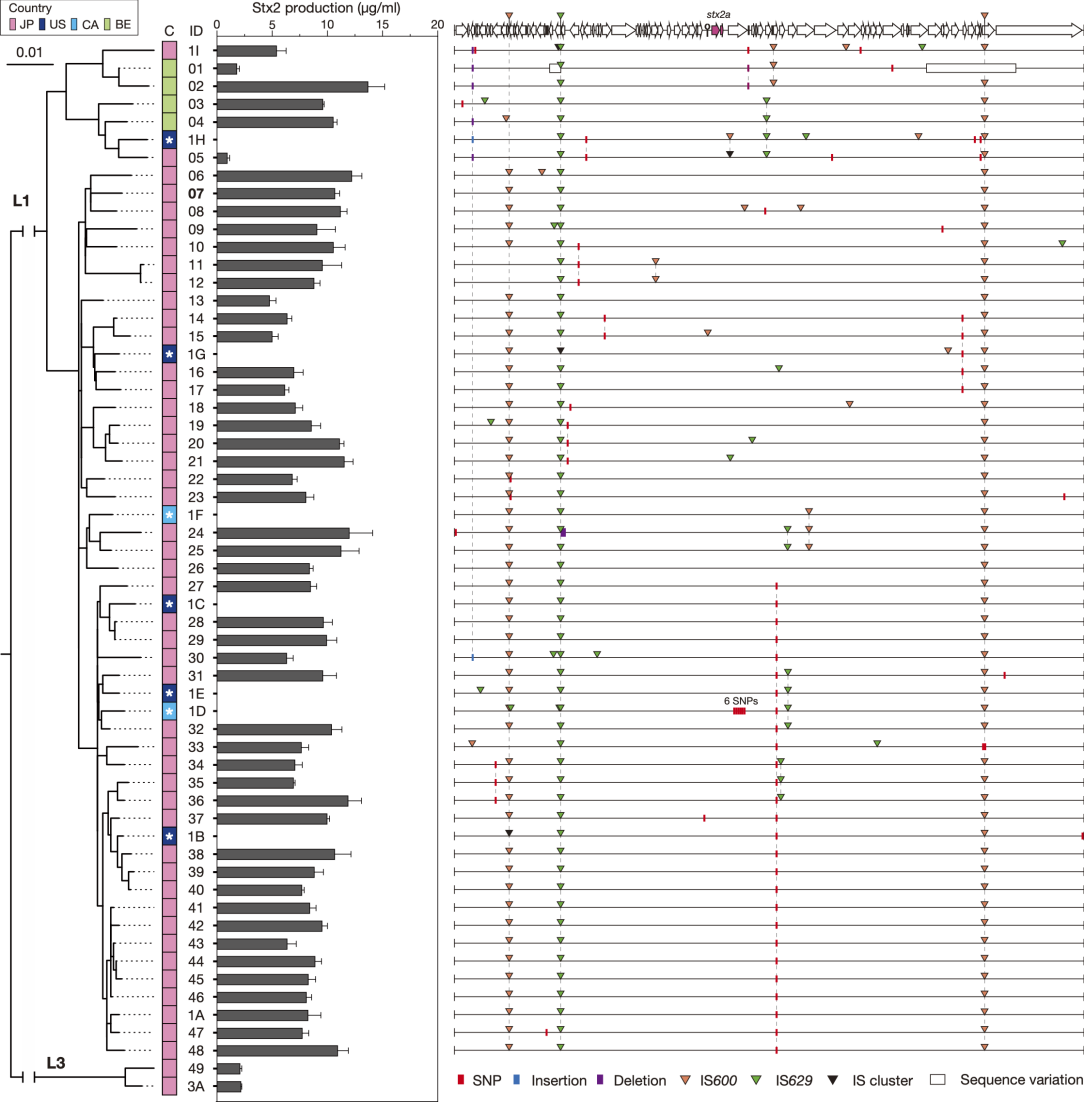
Variation in the Stx2 production level and Stx2a phage genome sequence between O121:H19 strains. The left panel shows an ML tree of 52 strains used in this analysis and seven completely sequenced lineage L1 strains. The tree was constructed based on the recombination-free SNPs (3439 sites) identified on the chromosome backbone (4010134 bp in total), and the geographic information and strain IDs of each strain (see Table S3 for details) are also shown. The seven complete genomes downloaded from the public database are indicated by an asterisk. In the centre panel, the MMC-induced Stx2 production levels are shown by the mean values with standard deviations of biological triplicates. The experimental conditions for Stx2 production measurement were optimized using four strains (see Fig. S1 for details). The right panel schematically presents the alignment of genome sequences of Stx2a phages of the strains belonging to L1. The sequences were aligned with the IS-free genome sequence of the Stx2a phage of strain 4151 (ID07) shown at the top. All these phages are integrated into the *argW* gene. SNPs, indels, and IS insertions detected at the same site are indicated by broken lines. The sequences of the two regions depicted by open rectangles were highly divergent and unable to be aligned with the reference sequence, and their genetic structures are shown in Fig. S8. The Stx2a phages of two lineage L3 strains were integrated into *yecE*, and their genome sequences were different from those of the Stx2a phages of L1 strains. See Fig. S8 for their genome sequences and comparison with the Stx2a phages in lineage L1. JP: Japan, US: United States of America, CA: Canada, BE: Belgium. Bar, the mean number of nucleotide substitutions per site.

To investigate the variation in Stx2 production level in O121:H19, we selected 52 *stx2a*-positive strains from the 143 strains sequenced in this study (Table S3). This strain set comprised 50 L1 and two L3 strains and included three strains completely sequenced in this study (two L1 and one L3 strain). Among the six *stx2*-positive strains belonging to L3 (Fig. 2b), only two strains were available for this analysis. As the 52 strains selected were isolated from Japan or Belgium, we first constructed an ML tree using the core genome sequences of the 52 strains and seven previously completely sequenced L1 strains and confirmed that this strain set largely represented the entire phylogeny of lineage L1 (Fig. 4). Then, the Stx2 production levels of the 52 strains were determined.

As shown in Fig. 4, the production levels of the L1 strains examined were in the range of 4.8–14 µg ml^−1^, except for two strains (1.8 µg ml^−1^ and 0.95 µg ml^−1^ in strains ID01 and ID05, respectively, in Fig. 4). In contrast, both L3 strains showed lower Stx2 production levels (2.1 and 2.2 µg ml^−1^, respectively) than L1 strains.

Next, to examine the relationship between the variations in Stx2 production level and the genomic diversity of Stx2 phages, we determined the sequences of Stx2 prophages of 49 strains other than the three completely sequenced strains. As expected from the analysis of complete genomes (Fig. 2), all L1 strains contained Stx2a phages at *argW,* while those of the two L3 strains were found at *yecE* (Table S3). The Stx2a phages of the L1 strains were 65.0–71.4 kb in length, whereas those of the L3 strain were 44.2 kb and 46.6 kb, respectively. The genomic structures of Stx2a phages of L1 and the L3 strains also clearly differed; those of L1 strains were short-tailed phages, as they shared similar late genes with the Stx2a phages of O157 strains Sakai [15] and EDL933 [62], but those of L3 strains were long-tailed phages having late genes similar to phage lambda.

The sequences of the Stx2a phage of L1 strains were highly conserved, except for that of a Belgian strain (ID01), with only a few SNPs/indels and variations in IS insertions, and the distribution of these SNPs and IS insertions followed the phylogeny of host strains (Fig. 4). In the Stx2a phage of the ID01 strain, replacement of two segments occurred (Fig. S8). These findings support the aforementioned notion that the Stx2a phage was acquired by a common ancestor of L1 and stably maintained in this lineage and are consistent with the observation that there is no marked difference in the levels of Stx2 production among the L1 strains except that the two strains (ID01 and ID05) showed lower Stx2 production levels. In one of the two strains (ID01), a part of the early region encoding gene *n* was replaced as mentioned above (Fig. S8). As Stx2 production has been related to phage induction [63], the low level of Stx2 production by this strain may be related to this replacement. In contrast, the genome of the Stx2a phage of another exceptional strain (ID05) was almost the same as those of other L1 strains. However, we detected three SNPs and an IS insertion unique to this Stx2a phage (Fig. 4), suggesting the possibility that either of these SNPs or the IS insertion may be associated with the lower Stx2 production level of this strain.

The lower Stx2 production levels of the L3 strains may be related to the fact that similar to Stx2a phages of L1 strains, those of L3 strains were lambda-like long-tailed phages, but we cannot exclude the possibility that some difference in the genetic background of host strains also affects the Stx2 production level of this lineage. A possibility that some other *stx2*-positive L3 strains produce higher amounts of Stx2 also can not be excluded. Thus, more L3 strains need to be analysed to make a conclusion on the Stx2 production level of L3 strains. It should also be mentioned that the Stx2a phages of the two L3 strains analysed here showed low sequence similarity to each other (<95 % nucleotide identity in >60 % of the genome), suggesting the independent acquisition of these Stx2a phages by the two L3 strains or intensive recombination of their Stx2a phages with other lambda-like phages [64]. An Stx1a phage found at the *ssrA* locus of strain 1A (Fig. 2), one of the two completely sequenced L3 strains, was also a lambda-like long-tailed phage.

### Temporal analysis of O121:H19

Finally, we performed a temporal analysis of O121:H19 strains by Bayesian coalescent analysis [53]. As shown in Fig. S9, the maximum clade credibility tree indicated that the time to the most recent common ancestor (TMRCA) of the entire O121:H19 lineage was approximately 752 years ago (95 % highest posterior density: 655–858 years), and the separation of the four lineages (L1-L4) occurred during an approximately 130 year period (from 1269 to 1395). Considering the LEE-negative status of the outlier (strain NCTC9064), it was suggested that the common ancestor of O121:H19 acquired the LEE element 750–1000 years ago. As mentioned above, lineages L1 and L3 acquired virulence plasmids with different backbones (Fig. S6). The separation of L1 into multiple sublineages that are currently circulating worldwide was a relatively recent event (approximately 80 years ago). Considering the high conservation of Stx2a phage at *argW* in lineage L1, it is most likely that the acquisition of Stx2a phage by the common ancestor of this lineage occurred shortly before this separation.

## Conclusion

Through a global WGS analysis of O121:H19 STEC, we identified four lineages. The major and second major lineages (named L1 and L3, respectively), mainly comprising ST655 strains, are both circulating worldwide. Analyses of major virulence-related genes and plasmid replicons among the entire O121:H19 lineage and detailed analysis of 11 complete genomes belonging to L1 and L3 revealed the acquisition of the LEE by the common ancestor of O121:H19, the independent acquisition of virulence plasmids by lineages L1 and L3, and a notable difference in the prophage pool and prophage-encoded T3SS effectors between lineages L1 and L3. While notable diversity in the prophage repertoire was observed in lineage L3, prophages of lineage L1 showed strikingly high conservation. Consistent with this, L3 strains contained long-tailed Stx2a phages with markedly different genomes, but L1 strains contained highly conserved short-tailed Stx2a phages, which were most likely acquired by the common ancestor of L1 and has been stably maintained in this lineage. Importantly, L1 strains produced much higher levels of Stx2a than L3 strains. These findings provide a global phylogenetic overview of O121:H19 STEC and notable interlineage differences within this important but understudied STEC.

## Supplementary Data

Supplementary material 1

Supplementary material 2
