# Supplementary material for: The global population structure and evolutionary history of the acquisition of major virulence factor-encoding genetic elements in Shiga toxin-producing Escherichia coli O121:H19
Source: Microb Genom. 2021 Dec 8;7(12):000716. doi: 10.1099/mgen.0.000716 (PMC8767318; doi:10.1099/mgen.0.000716)
Supplement: Supplementary material 1 [file mgen-7-0716-s001.pdf]

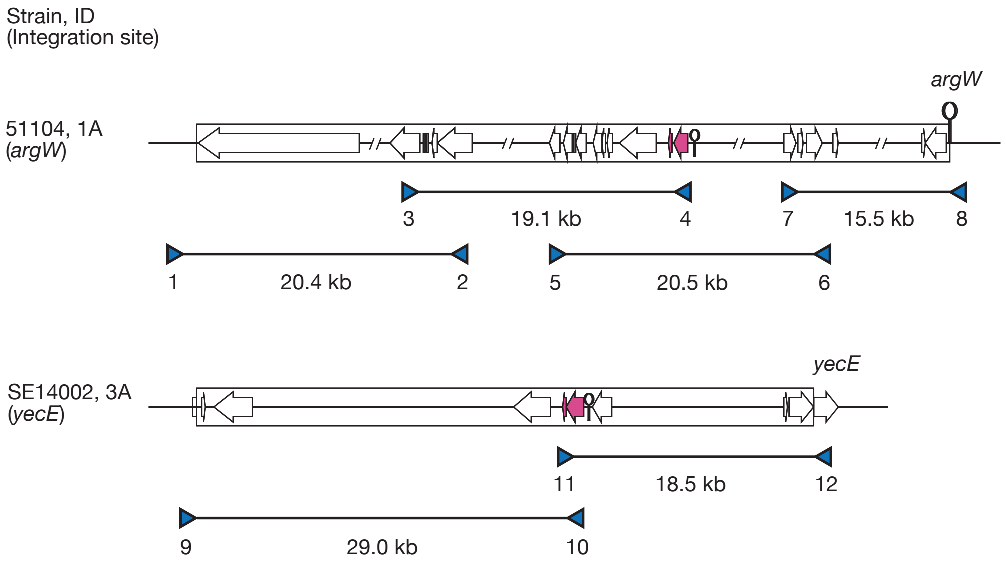

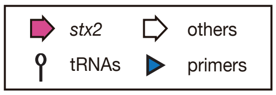

Primer	Sequence
1	CTTATTGCACTGGGTGACAC
2	CACTGTCAGTGCTGAAGAGG
3	CAACGGGACTACGGATCAC
4	GAATCAGCAATGTGCTTCCG
5	GTCGGATAGTTCATAGCGTC
6	CAGGGATCAGAACTGCATG
7	GTACTCATTGAGCCTAACAC
8	GCAATTAGCCCTTAGGAGG
9	TTACATCGGTCTACCGCAATGGTC
10	GAATCAGCAATGTGCTTCCG
11	TACCTTTAGCACAATCCGCCGC
12	TGTGTGGACGTGCTGCATGAAC

Fig. S1 Sequence determination of Stx2a phage genomes.

The long PCR-based sequencing strategy of Stx2a prophage genomes is shown. PCR primers were designed based on the sequences of the Stx2a phages found in the completely sequenced strains 51104 and SE14002. The Stx2a prophage region in each strain was divided into four or two segments and amplified by long PCRs. The PCR products obtained were subjected to Illumina sequencing to determine the sequence of the entire prophage region.

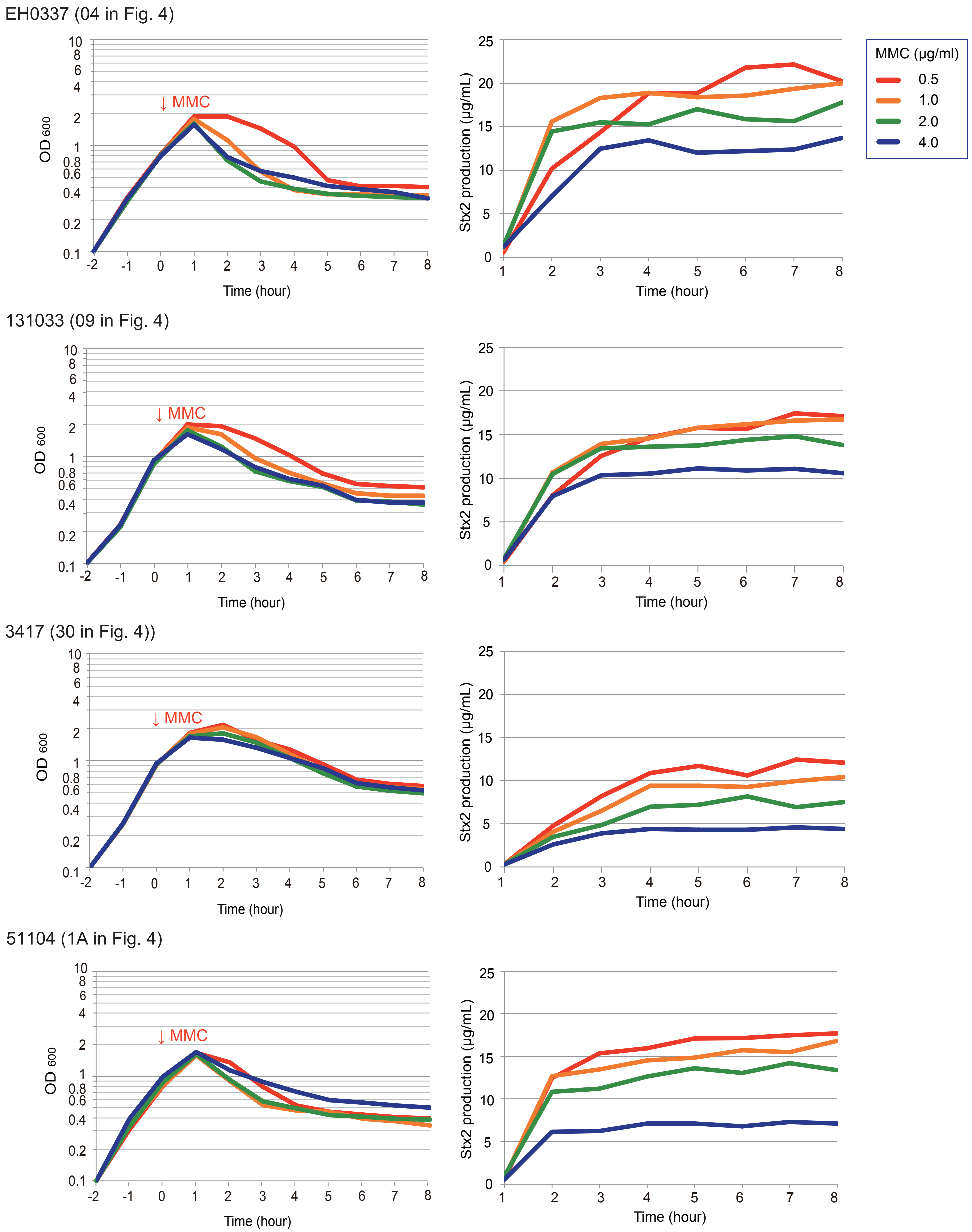

Fig. S2 Optimization of the Stx2 production assay.

Lysis curves (left) of four O121:H19 strains after mitomycin C (MMC) treatment and the Stx2 concentrations of cell lysates obtained at each time point (right) are shown. Bacterial cells were inoculated into 40 mL of LB broth at a cell concentration of 0.1 OD_{600} (optimal density at 600 nm) and grown to mid-log phase at 37°C with shaking. MMC was added to the culture at final concentrations of 0.5 µg/ml, 1.0 µg/ml, 2.0 µg/ml or 4.0 µg/ml. After the addition of MMC, the OD_{600} of each culture was measured every hour for 8 hr, and 100 µL of the culture was collected at each time point to prepare cell lysates. The Stx2 concentration in the lysate was determined by sandwich ELISA (n=1). As the nearly maximum cell lysis and the highest Stx2 concentration were observed, in most cases, after 6 hr of incubation with 0.5 µg/ml MMC, this condition was employed as the optimal condition for the Stx2 production assay of O121:H19 strains.

(a)

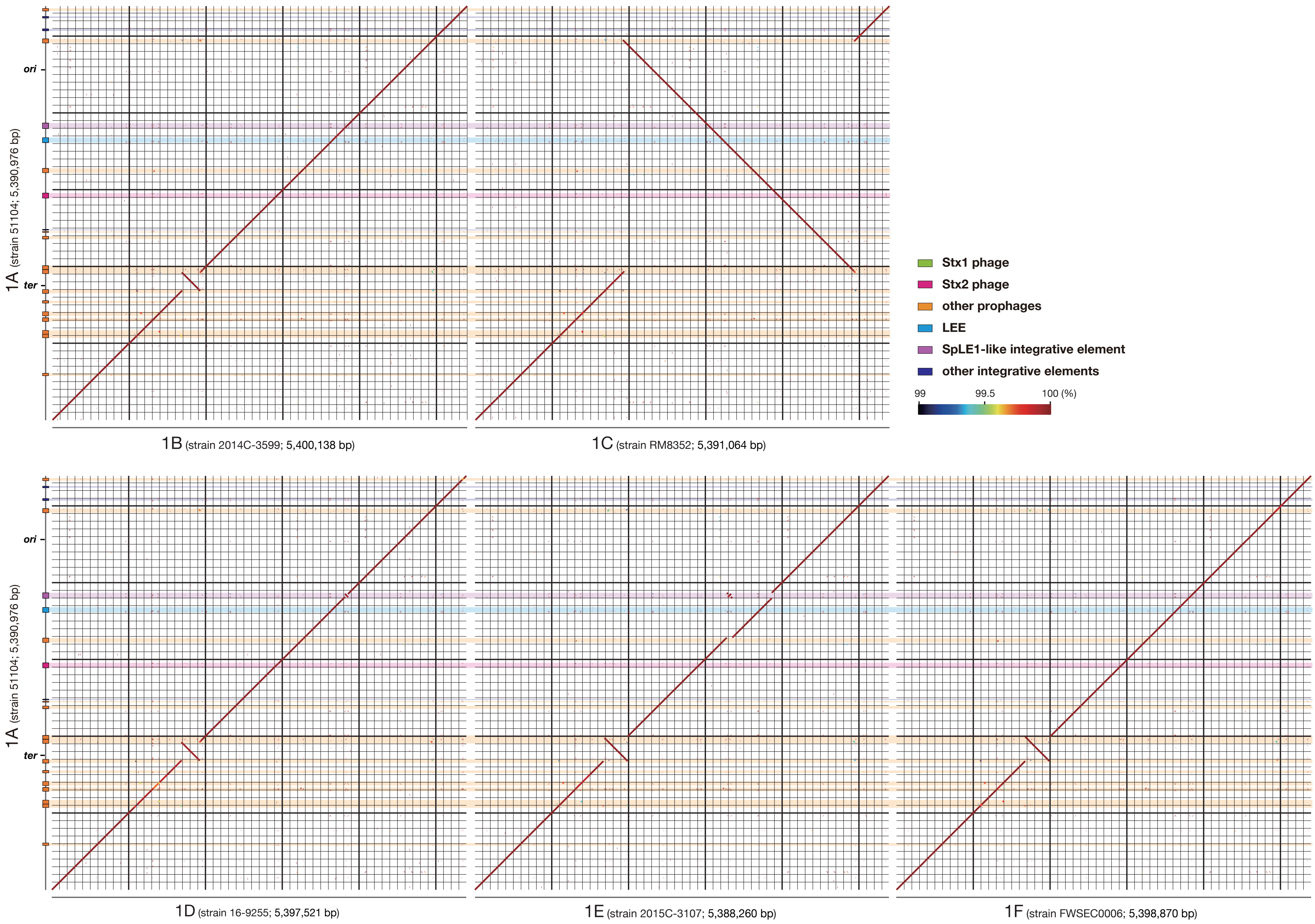

ori
ter
1A (strain 51104; 5,390,976 bp)
1B (strain 2014C-3599; 5,400,138 bp)
1C (strain RM8352; 5,391,064 bp)
1D (strain 16-9255; 5,397,521 bp)
1E (strain 2015C-3107; 5,388,260 bp)
1F (strain FWSEC0006; 5,398,870 bp)
Stx1 phage
Stx2 phage
other prophages
LEE
SpLE1-like integrative element
other integrative elements
99
99.5
100 (%)

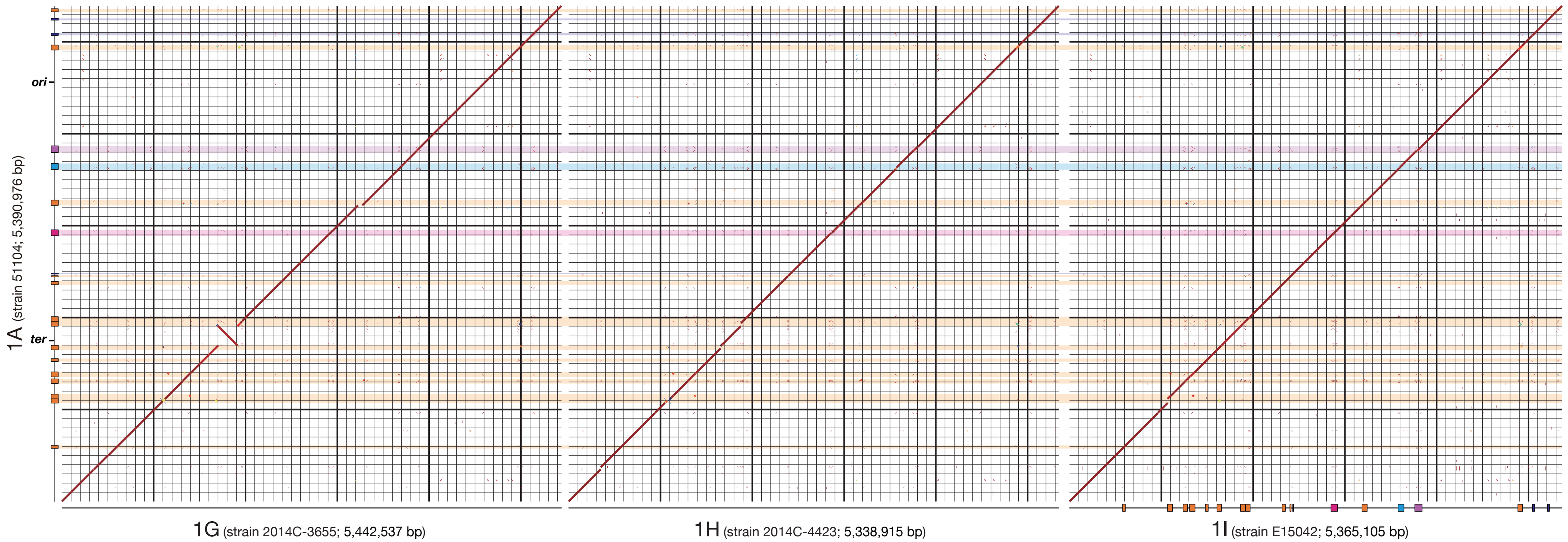
ori
ter
1A (strain 51104; 5,390,976 bp)
1G (strain 2014C-3655; 5,442,537 bp)
1H (strain 2014C-4423; 5,338,915 bp)
1I (strain E15042; 5,365,105 bp)

(b)

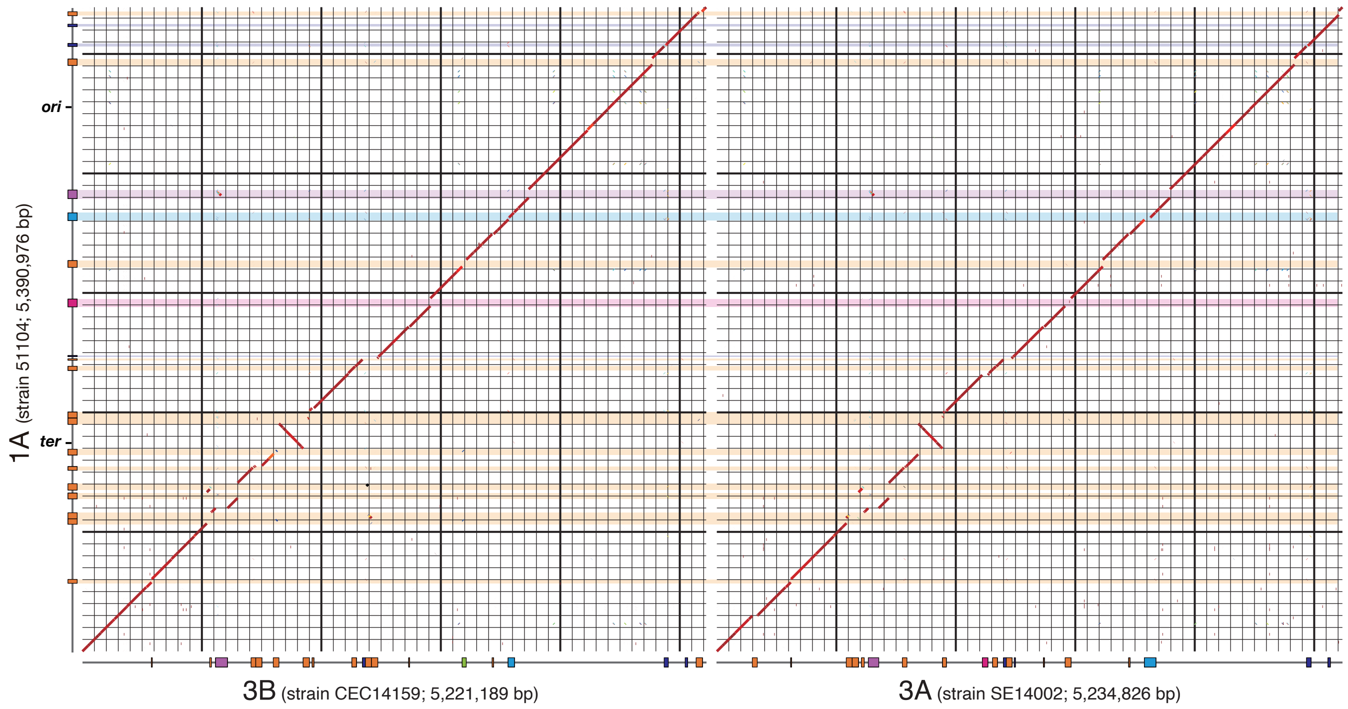
ori
ter
1A (strain 51104; 5,390,976 bp)
3B (strain CEC14159; 5,221,189 bp)
3A (strain SE14002; 5,234,826 bp)

(c)

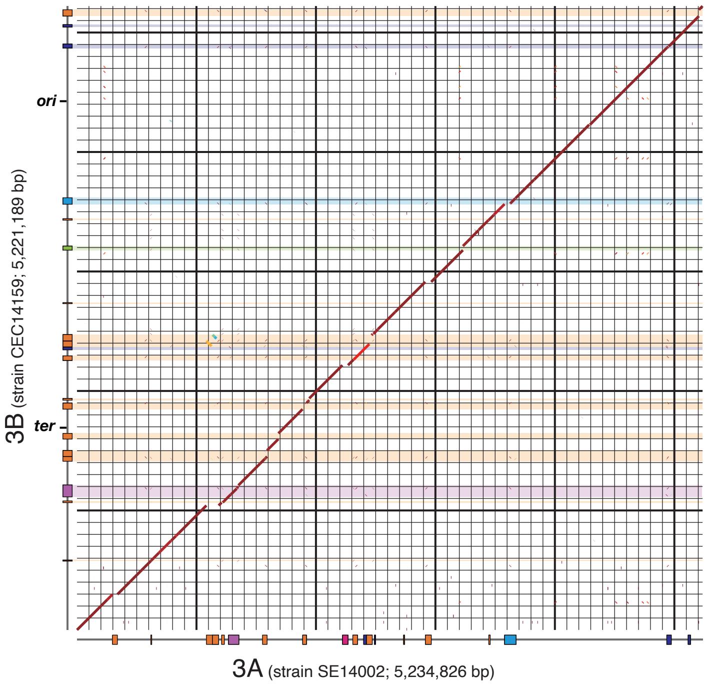
ori
ter
3B (strain CEC14159; 5,221,189 bp)
3A (strain SE14002; 5,234,826 bp)

Fig. S3 Comparison of the chromosome sequences of 11 completely sequenced O121:H19 strains.

Dot plot matrixes of chromosome sequences between 11 completely sequenced strains are shown. Strain 1A and eight L1 strains (panel a), strain 1A and two L3 strains, 3A and 3B (panel b), and two L3 strains, 3A and 3B (panel c), were compared in each panel. The positions of prophages and integrative elements on the chromosomes of strains 1A and 3B are indicated by shading with different colours (green, Stx1 phage; magenta, Stx2 phage; orange, other prophages; cyan, LEE; purple, SpLE1-like integrative elements; blue, other integrative elements). Only the sequences with >99% identity are shown with a heat map.

(a)

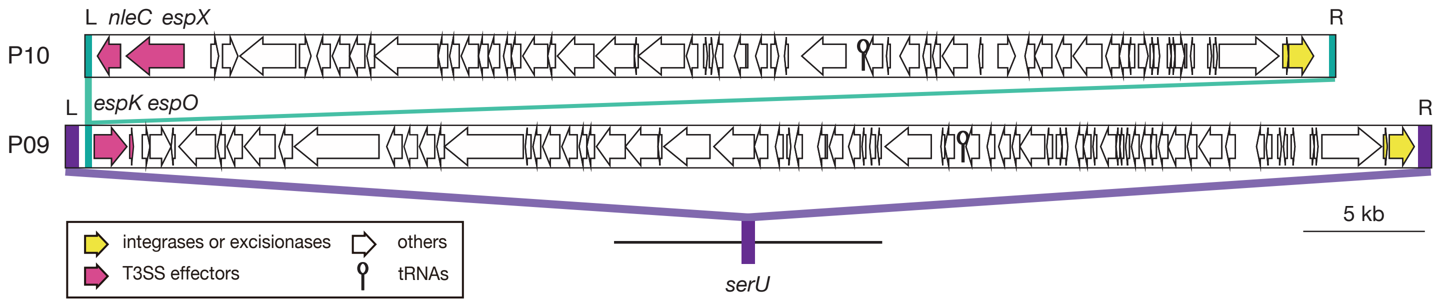

(b)

strain (ID)	prophage or tRNA	att site	1 … 61
Sakai	Sp14	*attP*	TGGCGGAGAGA--GGGGGATTTGAACCCCCGGTGGAGTTGCCCCCACTCCGGTTTTCGAGACC
CEC14159 (3B)	P09	*attL*	***********GG*******************A**********T*******************
		attR	***********__**
MG1655	*serU*	*attB*	***********__*******************A**********T*******************

(c)

strain (ID)	prophage	*att* site	1 … 26
SE14002 (3A)	P10	*attB* in prophage	ATGAGCGA-CCTAATCCATGCATGACG
CEC14159 (3B)	P10	*attL*	********_******************
		attR	*******A*T*****************

Fig. S4 A prophage integrated into an *attB* site in the prophage integrated into the *serU* gene.

(a) The integration of a prophage (P10) into the prophage genome integrated into the *serU* (P09) in strain CEC14159 (ID: 3B). The structures of prophages P09 and P10 are drawn to scale. P10 has been integrated at the *attB* site located upstream of a T3SS effector gene (*espK*) in the P09 genome. (b) The *att* sequences of prophages P09. The putative *attL/R* sequences of P09 were aligned with the *attP* sequence of the Sp14 prophage of the O157: H7 strain Sakai [Hayashi T, *et al*. *DNA Res* 2001;8:11-22] and the putative *attB* sequence, the sequence of the corresponding region in the *serU* gene of MG1655 (accession No. NC_000913) where no prophage was integrated. (c) The *att* sequences of prophage P10. The putative *attL/R* sequences of P10 were aligned with the putative *attB* sequence in prophage P09. As the MMC-induced excision (and replication) of P10 was not confirmed, the *attP* sequence was not be determined. The sequence of a corresponding region in the *serU*-integrated prophage (P10) of strain SE14002 (ID: 3A) that belongs to the same lineage (L3) as that of strain CEC14159 (ID: 3B) was used as the putative *attB* sequence. Note that the *attB* sequence in prophage P10 of strain SE14002 (ID: 3A) was different from the three *attB* sequences in previously reported prophage genomes [Nakamura K, *et al*. *PLoS Pathog* 2021;17:e1009073.]

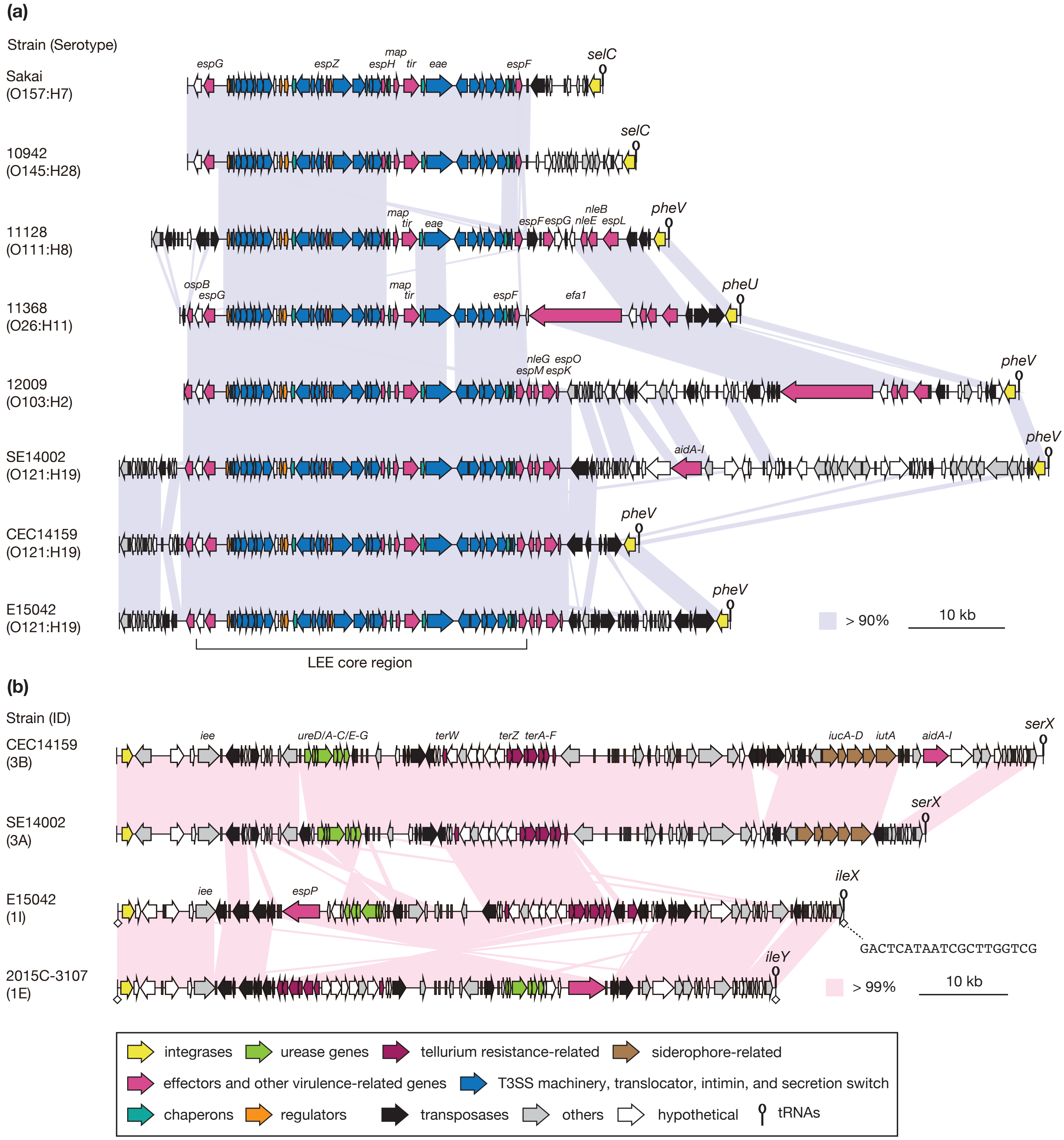

Fig. S5 LEE elements and SpLE1-like integrative elements in the completely sequenced O121:H19 strains. (a) Comparison of the LEE elements in three completely sequenced O121:H19 strains with those in STEC strains with other major serotypes. The genetic structures of the LEE elements of three O121: H19 strains and STEC strains representing the other five serotypes are drawn to scale. The strain IDs of O121:H19 strains SE14002, CEC141159, and E15042 in this study were 3A, 3B, and 1I, respectively. As the genetic structure of LEEs of the nine completely sequenced lineage L1 strains are highly conserved, that of strain E15042 (ID; 1I) is shown. (b) Comparison of the SpLE1-like integrative elements of four completely sequenced O121:H19 strains. The genetic structures of these elements in the four O121: H19 strains are drawn to scale. Identical 20-bp *attL/R* sequences (GACTCATAATCGCTTGGTCG, indicated by diamonds) were identified for the SpLE1-like integrative elements of strains E15042 (ID; 1I) and 2015C-3107 (ID; IE). As the genetic structure of SpLE1-like elements of the completely sequenced lineage L1 strains are highly conserved except for that of strain strain 2015C-3107 (ID; 1E); those of strains E15042 and 2015C-3107 are shown. In each panel, homologous regions showing nucleotide sequence identities over the indicated thresholds are depicted by shading.

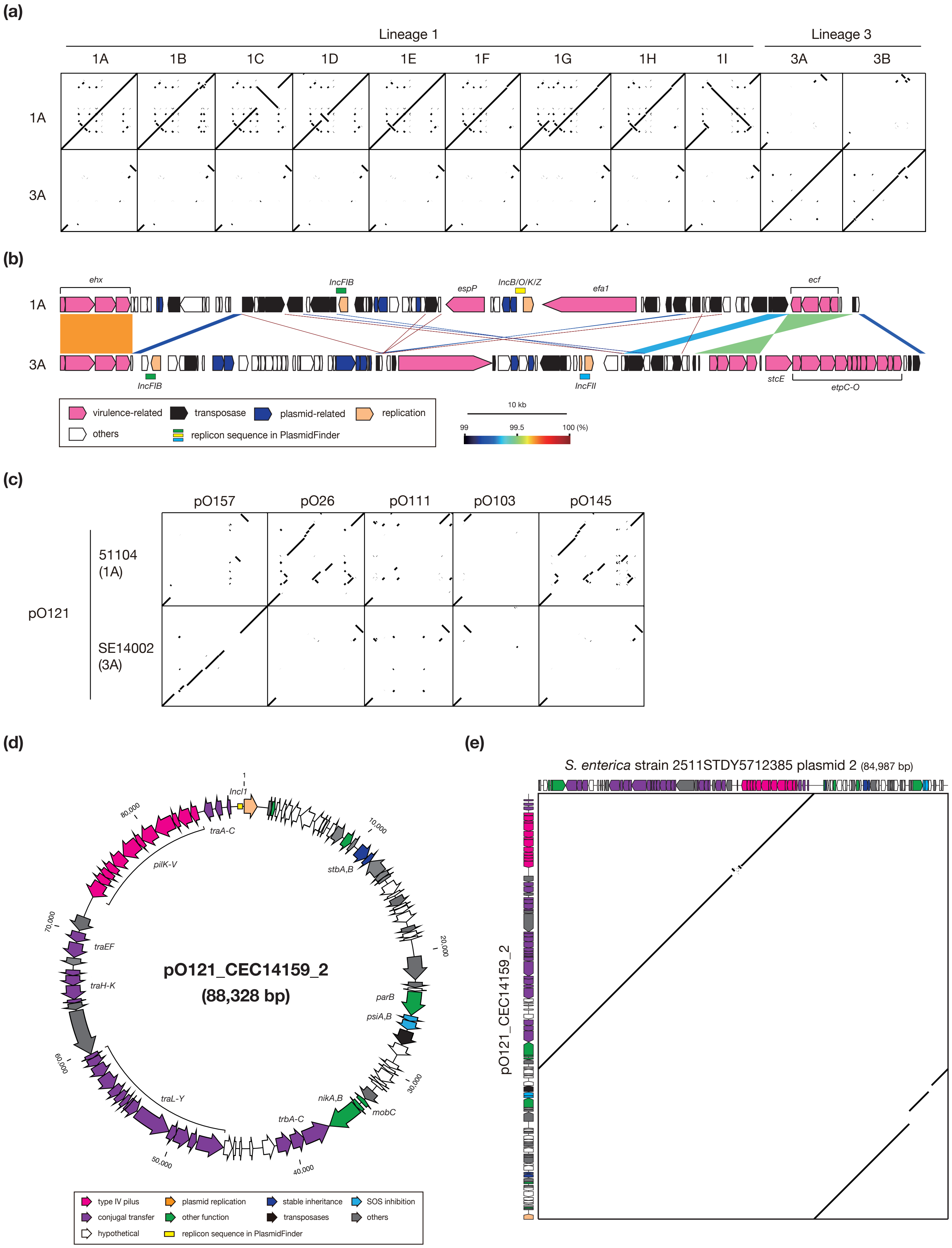
(a)
Lineage 1
Lineage 3
1A
1B
1C
1D
1E
1F
1G
1H
1I
3A
3B
(b)
ehx
IncFIB
espP
IncB/O/K/Z
efa1
ecf
IncFIB
IncFII
stcE
etpC-O
virulence-related
transposase
plasmid-related
replication
others
replicon sequence in PlasmidFinder
10 kb
99
99.5
100 (%)
(c)
pO157
pO26
pO111
pO103
pO145
51104 (1A)
pO121
SE14002 (3A)
(d)
IncI1
traA-C
pilK-V
stbA,B
traEF
traH-K
pO121_CEC14159_2 (88,328 bp)
parB
psiA,B
traL-Y
nikA,B
mobC
trbA-C
type IV pilus
plasmid replication
stable inheritance
SOS inhibition
conjugal transfer
other function
transposases
others
hypothetical
replicon sequence in PlasmidFinder
(e)
S. enterica strain 2511STDY5712385 plasmid 2 (84,987 bp)
pO121_CEC14159_2

Fig. S6 Plasmids found in the completely sequenced O121:H19 strains.

(a) Dot plot presentation showing the sequence similarities (>99% sequence identity) between the virulence plasmids of the 11 completely sequenced O121:H19 strains. The nucleotide sequences of the virulence plasmids of strain 51104 (1A) and strain SE14002 (3A) were each compared with those of other strains using the GenomeMathcher software. (b) Comparison of the genetic structures of virulence plasmids of strains 1A and 3A belonging to lineages L1 and L3, respectively. Homologous regions are depicted by shading with sequence identities presented by a heat map. “Plasmid-related” CDSs include genes related to conjugation, partitioning, and SOS inhibition. (c) Comparison of the virulence plasmids of two O121:H19 strains with those of STEC strains representing five other major serotypes. The nucleotide sequences of the plasmids of lineages L1 (strain ID; 1A) and L3 (strain ID; 3A) were compared with those of the O157:H7 strain Sakai (accession no. AB011549), O26:H11 strain 11368 (AP010954), O111:H8 strain 11128 (AP010963), O103:H2 strain 12009 (AP010959), and O145:H28 strain 10942 (AP019704), and the regions showing >99% sequence identity are shown by dot plots. (d) Circular map of an 88-kb nonvirulence plasmid (accession No. AP024477) identified in strain CEC14159 (ID; 3B) sequenced in this study. (e) Dot plot presentation showing the nucleotide sequence similarities (>95% sequence identity) between the nonvirulence plasmid of strain CEC14159 shown in panel (d) and the plasmid of *S. enterica* serovar Weltevreden strain 2511STDY5712385 (accession No. LN890525).

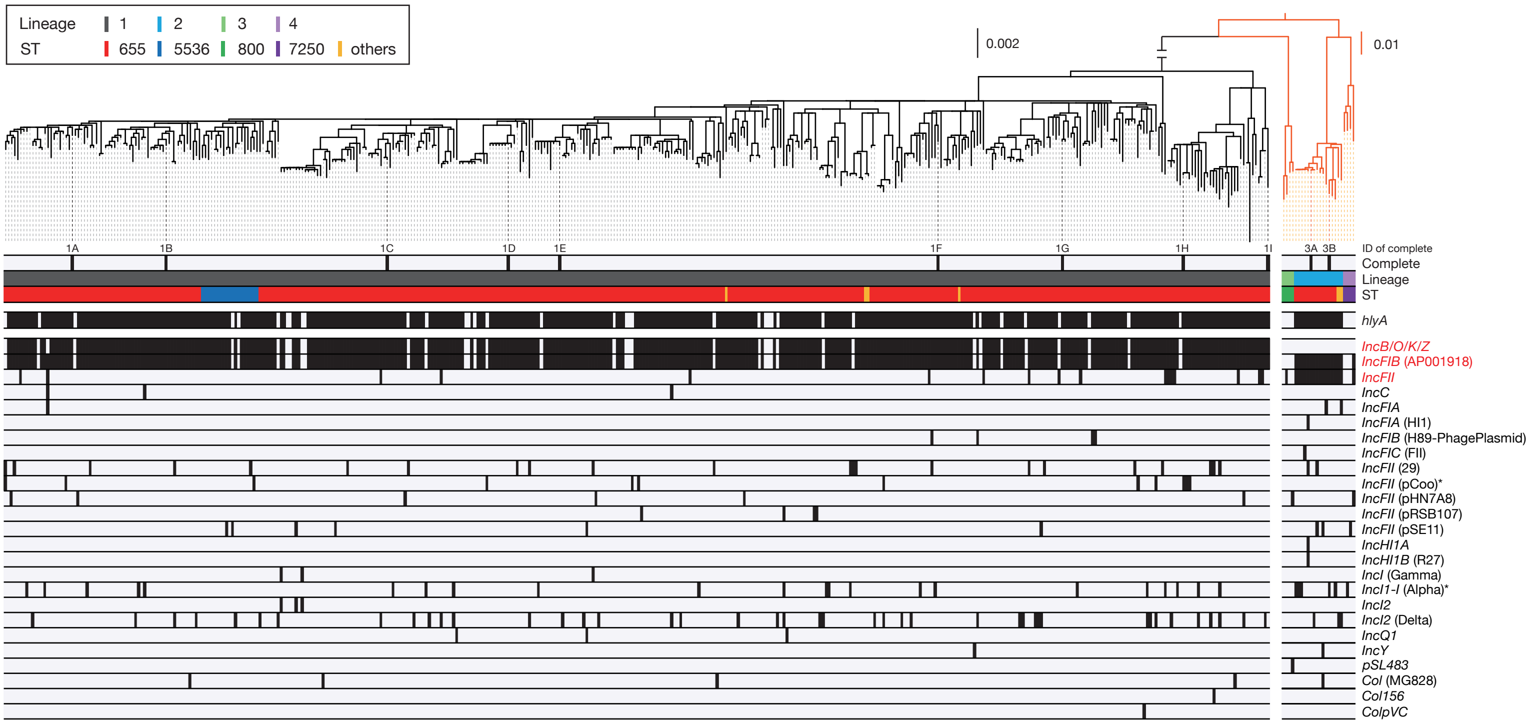

Fig. S7 The distribution of plasmid replicons in O121:H19.

The results of the plasmid replicon search by PlasmamidFinder were mapped to the ML tree (the same tree shown in Fig. 2b) containing 442 O121:H19 strains. The three replicons found in the virulence plasmids are indicated by red, and those of the two nonvirulence plasmids found in the 11 completely sequenced genomes are indicated by asterisks. Black and grey boxes indicate the presence and absence of each plasmid replicon, respectively. The distribution of the *hlyA* gene encoded by virulence plasmids is also shown.

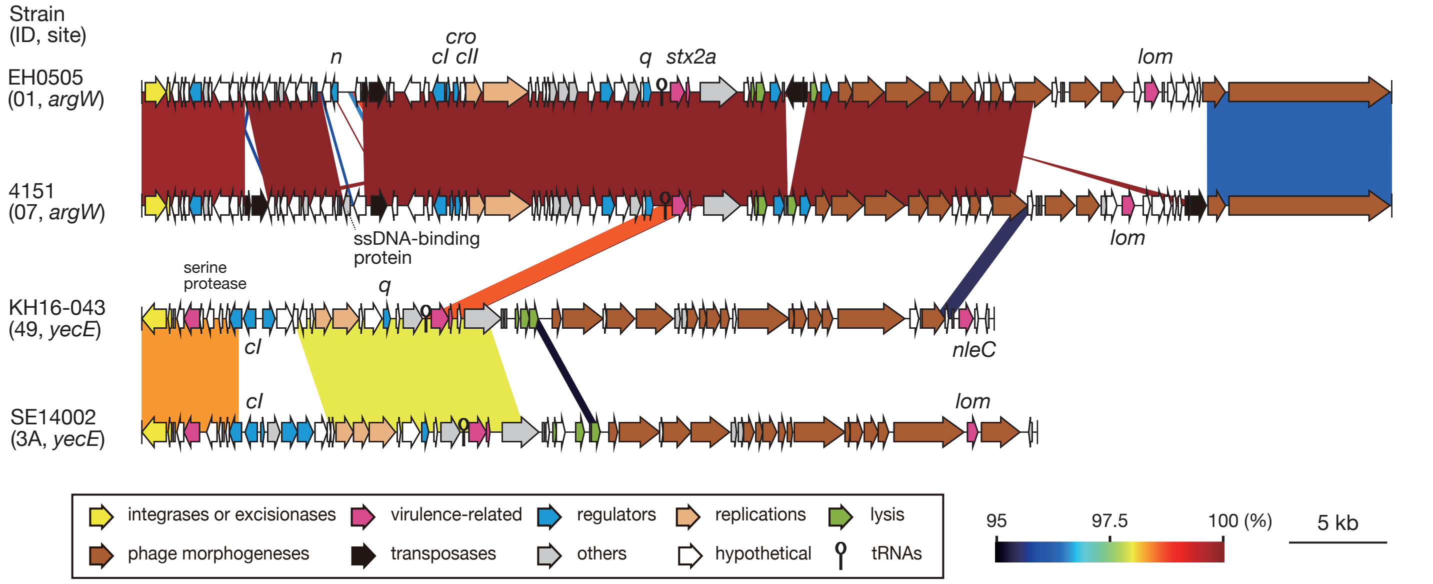

Fig. S8 The genomes of Stx2a phages in O121:H19 lineage L3.

The genetic structures of the Stx2a phages of strains EH0505 and 4151 belonging to L1 and strains KH16-043 and SE14002 belonging to L3 are drawn to scale. Stx2a phages of lineages L1 and L3 are integrated into *argW* and *yecE*, respectively. Sequence homology between Stx2a phages is shown by shading with a heat map. The Stx2a phage of 4151 shared almost identical genome sequences with the Stx2a phages of other lineage L1 strains (see Fig. 4 in the main text), but the sequences of two regions of the Stx2a phage of strain EH0505 were replaced by unrelated sequences, which encoded an *n* gene homologue and a part of tail genes, respectively. The Stx2a phages of the two L3 strains have genomes different from those of L1 strains. While both are lambda-like long-tailed phages, their genome sequences are clearly different.

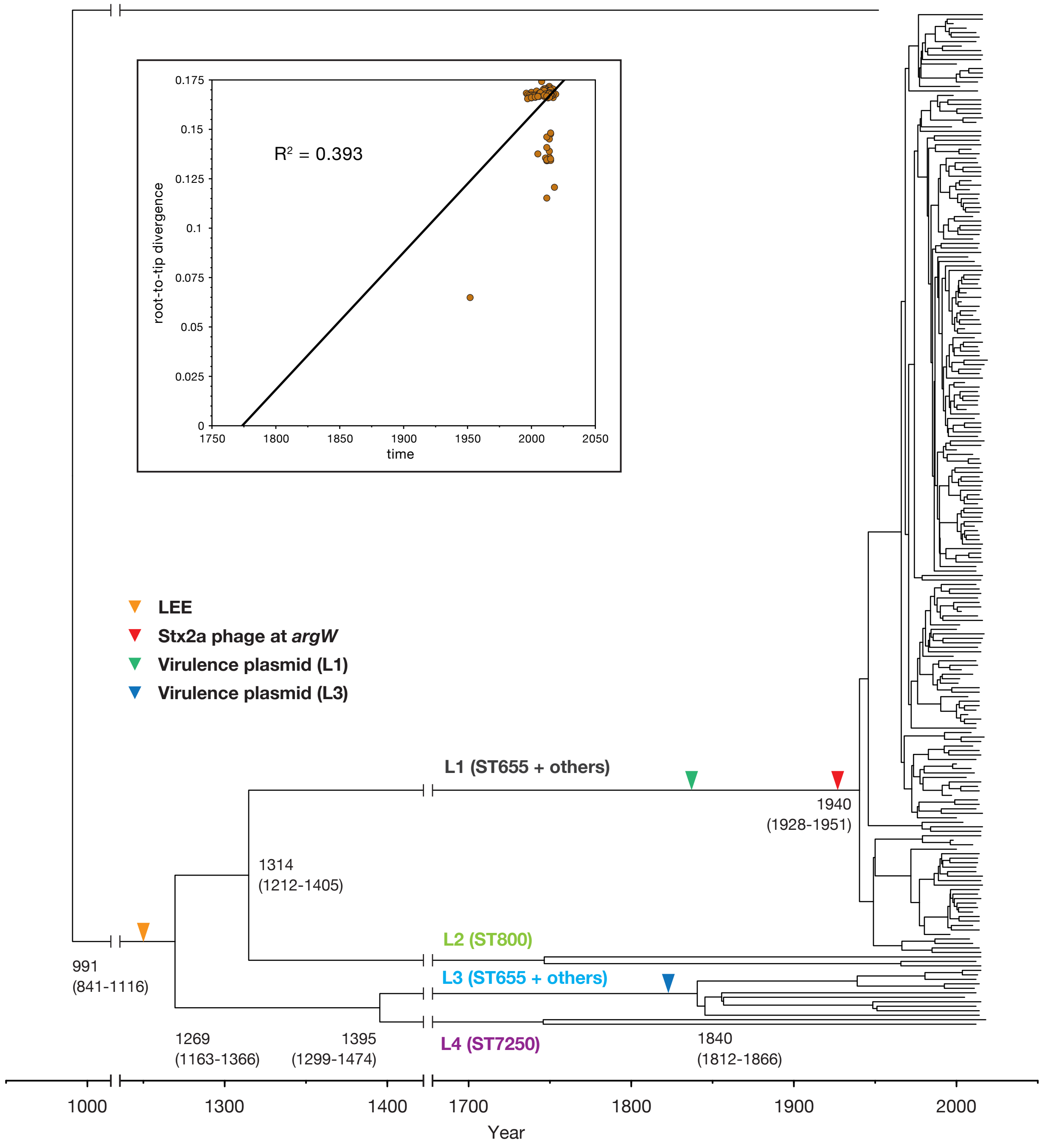

Fig. S9 A maximum clade credibility tree representing the evolutionary history of O121:H19 strains inferred by Bayesian evolutionary analysis.

The result of regression analysis of root-to-tip distance against sampling date is shown in the inset, indicating a positive correlation of genetic distance and sampling date. The time-calibrated phylogenetic tree was reconstructed using BEAST based on 6,923 concatenated recombination-free SNPs. The time to the most recent common ancestor (TMRCA) of each O121:H19 lineage is shown in the tree with the 95% highest posterior density (HPD) indicated in parentheses. Note that the overall topology of the tree is consistent with that of the ML tree shown in Fig. 2B in the main text. Acquisition events of genetic elements that encode key virulence factors of STEC O121:H19 are indicated in the tree by coloured triangles (orange, LEE; red, Stx2a phage; green, the virulence plasmid in L1; blue, the virulence plasmid in L3). Integration sites of Stx2a phages are also indicated.
